# On the Signs of Diseased Heart Afforded to the Hand Laid over the Precordia

**Published:** 1849-12

**Authors:** 


*On the Signs of Diseased Heart afforded to the Hand laid over the Precordia.* By Prof. Jaksch.—The purring tremor (*frémissement cataire*) perceived in certain affections of the heart, is felt most distinctly when the flat hand is laid over the part of the precordium corresponding to the point of the heart's impulse. When this peculiar tremor is dependent upon narrowing of the left auriculo-ventricular opening, it is perceived at the period corresponding to the diastole of the heart. Dr. Jaksch, however, states that he has observed it in cases of insufficiency of the aortic valves. For determining to which of these morbid conditions the tremor during the diastole is in any case due, he points out the following diagnostic signs: If it occurs when the impulse is feeble, the heart broad (as indicated by increased lateral dullness on percussion), and the second sound increased, it is dependant on narrowing of the left auriculo-ventricular opening; if, on the other hand, it coincides with an increased impulse, an hypertrophied left ventricle (as indicated by a tremulous impulse and increased dullness, in the longitudinal direction, on percussion,) and with absence of the second sound of the heart, it may be considered as most probably dependent upon imperfection of the aortic valves. In cases in which a contracted left auriculo-ventricular opening coincides with imperfect aortic valves, a purring tremor accompanying the diastole of the heart is sometimes observed coincidently with an increased impulse. The diagnosis of such cases is rendered sufficiently easy by the increased second sound audible in the pulmonary artery, the enlargement of the heart in its longitudinal and transverse direction, and the absence of the second sound from the aorta and the carotid arteries.

It is not uncommon, especially after pericarditis, that peculiar tremors or vibrations are produced within the pericardium, and may give to the hand laid over the region of the heart a sensation of grating, scraping, creaking, or even buzzing. The existence of previous pericarditis, the absence of change of form of the heart, the want of rythm, and the variableness of the morbid sound preclude much risk of error in the diagnosis.

By means of the hand laid over the precordium, Prof. Jaksch has perceived vibrations synchronous with the systole of the heart.

1. In cases of narrowing of the aorta from rigid semilunar valves.

2. In cases of dilatation, thinning, and relaxation of the portion of the aorta, immediately above the semilunar valve.

3. In aneurismal dilatation of the ascending aorta, accompanied by roughness of the internal surface of the vessel.

4. In some cases of true aneurism of the ascending aorta, with roughness of the orifice or internal surface of the same.

5. In a case in which numerous tendinous bands are stretched across the left ventricle near the orifice of the aorta.

6. In a case of perforation of the inner division of the bicuspid valve.

7. Inefficiency of the bicuspid valve, in consequence of rupture of some of the tendinous cords.

8. In narrowing of the ascending aorta.

The sounds dependent upon disease of the aorta are perceived most distinctly when the hand is placed in the middle of the sternum, and is thence carried upwards and to the right, in the direction of the aorta.—*Medical Gazette.*

